# Localized CCR2 Activation in the Bone Marrow Niche Mobilizes Monocytes by Desensitizing CXCR4

**DOI:** 10.1371/journal.pone.0128387

**Published:** 2015-06-01

**Authors:** Hosung Jung, Divakar S. Mithal, Jeong Eun Park, Richard J. Miller

**Affiliations:** 1 Department of Anatomy, Brain Research Institute, and Brain Korea 21 PLUS Project for Medical Science, Yonsei University College of Medicine, Seoul 120-752, South Korea; 2 Department of Molecular Pharmacology and Biological Chemistry, Northwestern University, 303 E Chicago Ave, Chicago, IL 60611, United States of America; University of Florida, UNITED STATES

## Abstract

Inflammatory (classical) monocytes residing in the bone marrow must enter the bloodstream in order to combat microbe infection. These monocytes express high levels of CCR2, a chemokine receptor whose activation is required for them to exit the bone marrow. How CCR2 is locally activated in the bone marrow and how their activation promotes monocyte egress is not understood. Here, we have used double transgenic lines that can visualize CCR2 activation *in vivo* and show that its chemokine ligand CCL2 is acutely released by stromal cells in the bone marrow, which make direct contact with CCR2-expressing monocytes. These monocytes also express CXCR4, whose activation immobilizes cells in the bone marrow, and are in contact with stromal cells expressing CXCL12, the CXCR4 ligand. During the inflammatory response, CCL2 is released and activates the CCR2 on neighboring monocytes. We demonstrate that acutely isolated bone marrow cells co-express CCR2 and CXCR4, and CCR2 activation desensitizes CXCR4. Inhibiting CXCR4 by a specific receptor antagonist in mice causes CCR2-expressing cells to exit the bone marrow in absence of inflammatory insults. Taken together, these results suggest a novel mechanism whereby the local activation of CCR2 on monocytes in the bone marrow attenuates an anchoring signalling provided by CXCR4 expressed by the same cell and mobilizes the bone marrow monocyte to the blood stream. Our results also provide a generalizable model that cross-desensitization of chemokine receptors fine-tunes cell mobility by integrating multiple chemokine signals.

## Introduction

Innate immunity provides rapid protection from potentially harmful infection, before more specialized acquired immunity develops against specific antigens. Molecules such as Toll-like receptors (TLRs), which are expressed by numerous cells and respond to a variety of potential threats initiate innate inflammatory responses by increasing secretion of inflammatory cytokines. Inflammatory cytokines then activate a cascade of cellular responses that ultimately result in recruitment of activated leukocytes to the site of infection.

One major class of inflammatory cytokines, the chemokines, are a class of small-secreted proteins, which play diverse roles in orchestrating leukocyte trafficking by activating chemokine receptors [1]. Some chemokines show developmentally controlled or constitutive expression profiles, whereas others are upregulated under pathological conditions [2]. The inducible chemokines, such as CCL2 (a.k.a. MCP-1), are responsible for coordinated leukocyte movements in response to microbial infection [3]. Peripheral monocytes circulating in the bloodstream are a heterogeneous population of leukocytes. They can be categorized into two groups: CCR2^+^ and CX3CR1^+^ [4]. CCR2^+^ monocytes, which also expressing a high level of the Ly6C surface antigen, are enriched in the bone marrow under normal circumstances, and targeted into inflamed tissues. For this reason, they are also called Ly6C (high) or the classical monocytes. In contrast, CX3CR1+ monocytes are recruited to non-inflamed tissues, and resemble resident macrophages.

CCL2 is not normally expressed at high levels, but its expression rapidly increases during inflammation [5]. CCL2 is rapidly released in the bone marrow [6, 7] and promotes emigration of the classical monocytes [8]. CCL2 can also guide the classical monocytes in the peripheral blood stream to the inflamed tissue, under some pathological conditions such as thioglycollate-induced peritonitis [9] and experimental autoimmune encephalomyelitis (EAE), an animal model of multiple sclerosis [7, 10]. During bacterial infection the activation of CCR2 on classical monocytes is required for them to exit the bone marrow, whereas it is dispensable for directed movements in the bloodstream toward the infected tissue [8]. CCR2 and CCL2 knockout mice therefore cannot mobilize classical monocytes upon bacterial infection and die because they cannot suppress bacterial growth [8]. How CCL2 acts as a mobilizing signal rather than a long-range chemoattractant cue is not understood.

CXCL12 (a.k.a. SDF-1), acting on its receptor CXCR4, anchors hematopoietic stem cells [11], B lineage cells and granulocytic precursors [12], and neutrophils [13] in the bone marrow. All chemokine receptors belong to the G protein-coupled receptor (GPCR) family. Activation of one GPCR can alter signalling of another GPCR in several different ways. For example, CCR2 cross-desensitizes *μ*-opioid receptors in peripheral sensory neurons and induces heightened sense of pain [14]. Therefore, we reasoned that CCR2 activated during the inflammatory response might attenuate the force that normally retains the monocytes in the bone marrow, which is provided by CXCR4-mediated signaling.

## Results

### 1. Stored CCL2 is released from stromal cells during inflammation

**Fig 1:**
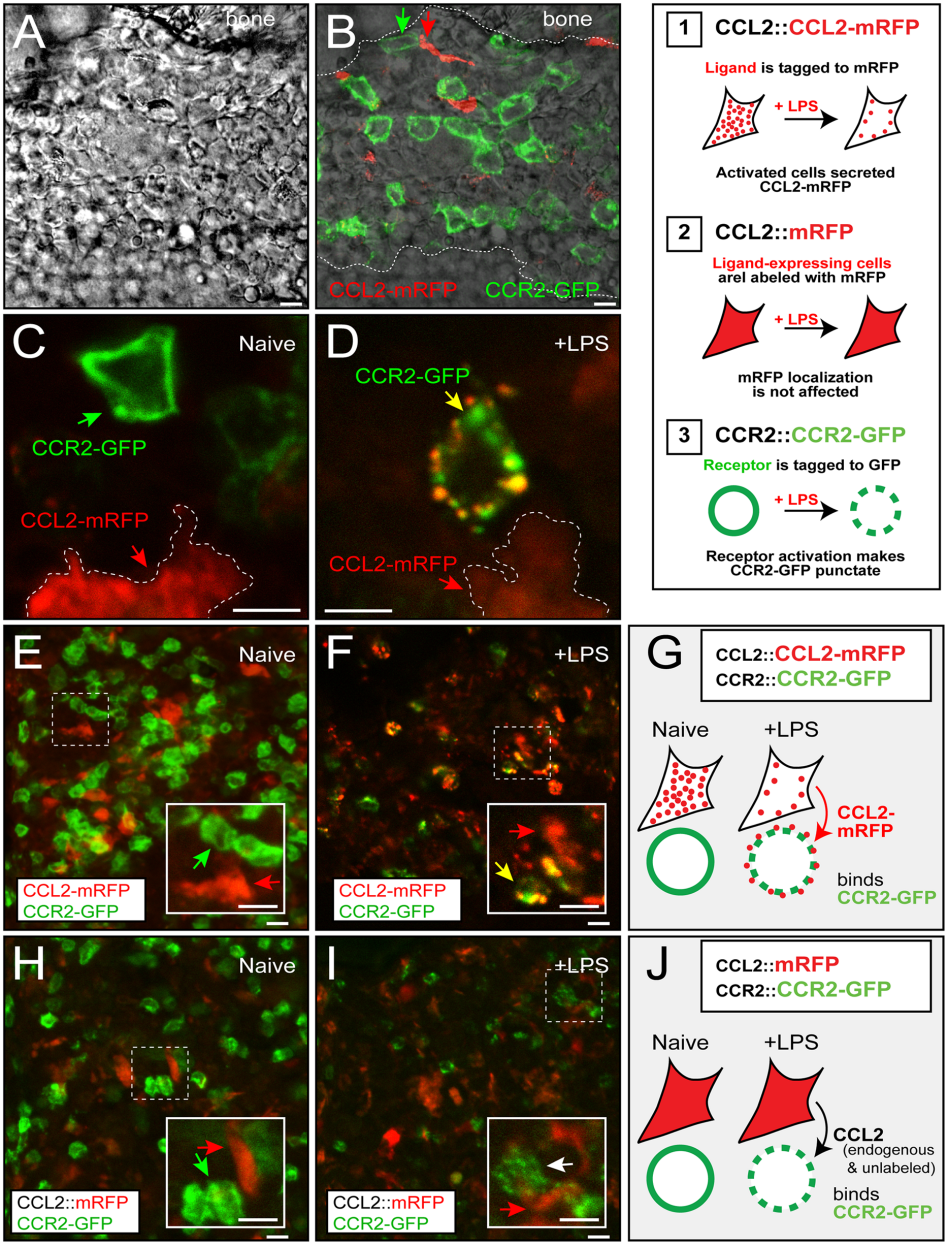
CCL2 acts on CXCR4-positive cells during inflammation in the bone marrow. (Inset) Transgenic reporter mice used in this study. 1) CCL2::CCL2-mRFP (CCL2 protein level reporter), 2) CCL2::mRFP (CCL2 transcriptional reporter), and 3) CCR2::CCR2-EGFP (CCR2 protein level reporter) (A-G) Bone marrow of CCL2::CCL2-mRFP; CCR2::CCR2-EGFP double transgenic mice. A differential interference contrast (A) and an overlaid fluorescent image (B) show CCL2-mRFP and CCR2-EGFP cells in close contact (arrows). (C-G) Under normal conditions, CCR2-EGFP mostly localizes to cell surface (green arrow). After LPS injection, most CCR2-EGFP cells are activated by CCL2, which is evident in granular CCR2-EGFP signals and endocytic vesicles containing both CCL2-mRFP and CCR2-EGFP (yellow arrow). (H-I) Bone marrow of CCL2::mRFP; CCR2::CCR2-EGFP double transgenic mice. After LPS injection, most CCR2-EGFP cells are activated by endogenous CCL2, which is evident in granular CCR2-EGFP (white arrow). Scale bars, 10 μm.

We previously established bacterial artificial chromosome (BAC)-transgenic mouse lines to label chemokines and chemokine receptors with fluorescent proteins at the protein level, and visualized CCR2 activation *in vivo* using the endocytosis of CCL2-CCR2 complexes as a readout, [7, 15]. Using CCL2::CCL2-mRFP;CCR2::CCR2-EGFP double transgenic mice, we examined where CCL2 and CCR2 proteins are expressed in the bone marrow under normal conditions (Fig 1A and inset). As expected, we observed that CCR2 is expressed at the surface of numerous monocytes in the bone marrow indicating that these receptors are not activated (Fig 1B, green arrow). Unexpectedly, we observed that CCL2, whose expression is known to be upregulated under pathological conditions, is also highly expressed by stromal cells under normal conditions (Fig 1B, red arrow). Intriguingly, the CCL2-positive stromal cells had elongated morphology and were almost always in direct contact with CCR2-positive monocytes (Fig 1B, green and red arrows), reminiscent of immunological synapse described for T cells and B cells in lymph nodes [16]. These results indicate that CCL2 proteins are stored in these stromal cells that are juxtaposed to CCR2-expressing monocytes, but are not released (because CCR2 was not internalized but remained on the cell surface of the monocyte).

**Fig 2:**
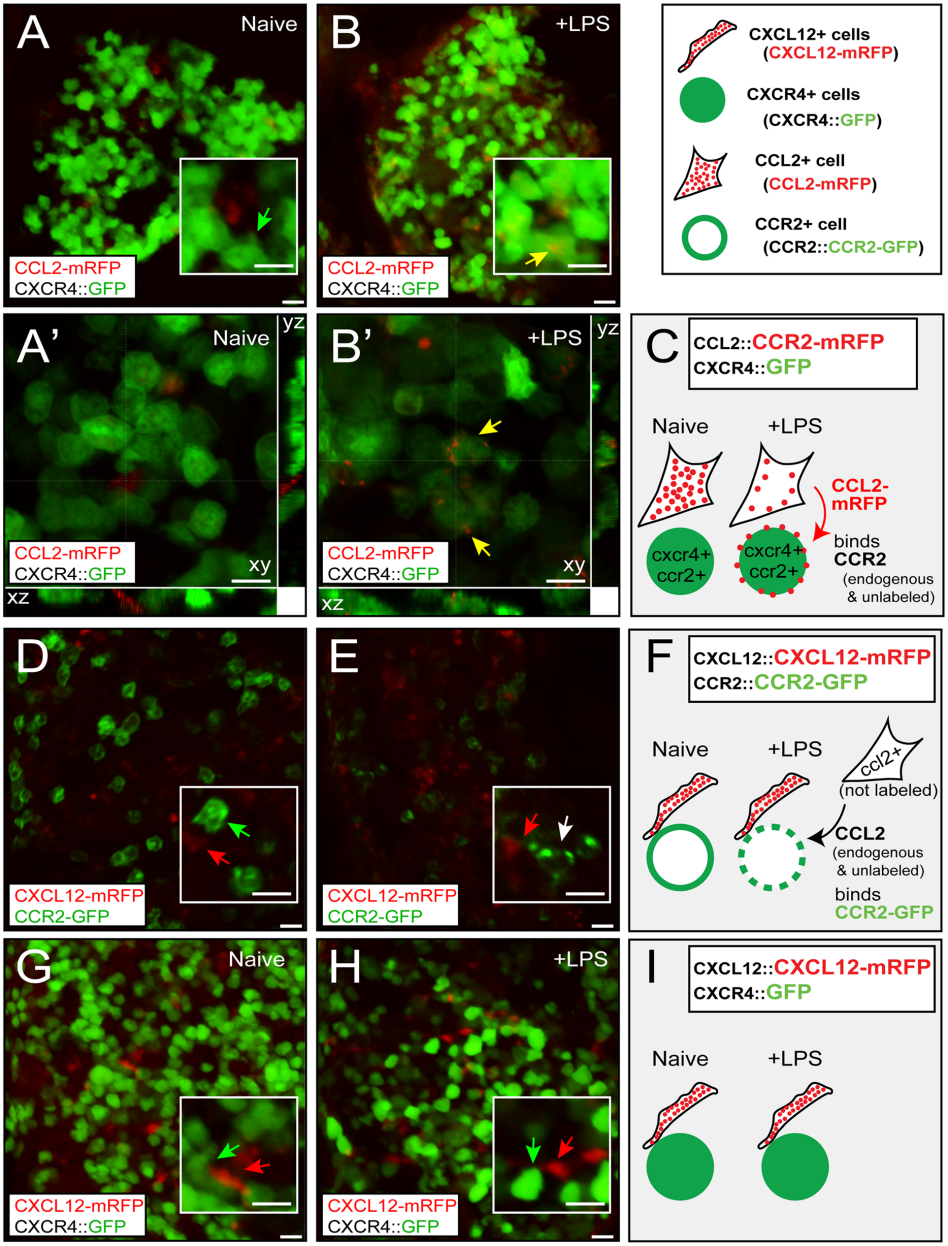
CCR2-positive cells are neighbored by CXCL12-positive cells in the bone marrow. (inset) Transgenic reporter mice used in this study. (A-C) Bone marrow of CCL2::CCL2-mRFP; CXCR4::EGFP double transgenic mice. Note that CXCR4::EGFP is a transcriptional reporter, and therefore EGFP localizes to the entire cell in which CXCR4 is expressed (C). Most CCL2-mRFP cells are in close proximity to CXCR4::EGFP cells (green arrow) under normal conditions. After LPS injection, these CXCR4::EGFP cells now contain CCL2-mRFP granules (yellow arrow), indicating endocytosis of CCL2-mRFP/CCR2 (unlabeled endogenous CCR2) (B’). (D-F) Bone marrow of CXCL12::CXCL12-mRFP; CCR2::CCR2-EGFP mice. CCR2-postive cells (green arrow) are often in contact with CXCL12-positive cells (red arrow). Upon LPS injection, CCR2 is activated (white arrow: granular CCR2-EGFP signal) independently from CXCL12 signaling (because CXCL12-mRFP is not endocytosed by CCR2 positive cells). (G-I) Bone marrow of CXCL12::CXCL12-mRFP; CXCR4:EGFP mice. CXCR4 activation, which could have been visualized by CXCL12 (red arrow) endocytosis by CXCR4-positive cells (green arrow), is not increased by LPS injections. Scale bars, 10 μm.

We induced an acute inflammatory response in these mice an intraperitoneal injection of lipopolysaccharide (LPS). We observed a striking change in CCL2 and CCR2 subcellular localization before and after an LPS injection. First, CCL2 proteins in elongated stromal cells became less distinct (Fig 1C and 1D, red arrow), possibly because many CCL2 had been secreted and less remained in these cells. We quantitated the RFP content per cell by quantitative fluorescence imaging, and found that LPS injection significantly decreased CCL2-mRFP per cell indicating its release (Fig 1E and 1F; S1A Fig). Because CCL2 is known to be upregulated by inflammatory insults [1], we asked whether LPS increases the number of CCL2-expressing cells in the bone marrow. For this, we used a different transgenic reporter CCL2::mRFP (CCL2 transcriptional reporter), in which CCL2-positive cells express mRFP only but not CCL2-mRFP fusion proteins (Fig 1 inset). For this reason, the pattern of mRFP signal in the CCL2-expressing cells is not affected by LPS, which allows consistent cell counting (S1B Fig). LPS injection led to an approximately 20% increase in the number of CCL2-mRFP expressing cells (S2A Fig; Fig 1H and 1I). Therefore, although many cells in the bone marrow express CCL2 under normal conditions, more cells begin to express it during inflammation. Second, most CCR2 appeared punctate, which localized both to the cell surface and cytosol of the monocytes (Fig 1D, yellow arrow). Overall, the number of CCR2-positive cells became smaller after the LPS injection, possibly because many of them left the bone marrow (Fig 1E–1G; S2B Fig). Importantly, the monocytes that were still in the bone marrow contained both CCL2 and CCR2 proteins (Fig 1D and 1F, yellow arrow). Because no cells contained both CCR2 and CCL2 under normal conditions (Fig 1C), we concluded that the CCL2 and CCR2 double positive puncta indicate ligand-receptor complexes that are endocytosed after CCR2 activation. CCR2 activation could be seen even when CCL2 is not fluorescently tagged, which was evident in punctate CCR2-EGFP (Fig 1H–1J; Fig 2D–2F). Taken together, these results indicate that CCL2 stored in the bone marrow under normal conditions is rapidly released during inflammation and activates CCR2 expressed by neighboring monocytes.

### 2. CCR2 activated monocytes express CXCR4

Next, we sought to ask whether CCR2-positive monocytes co-express CXCR4 and whether CCL2-CCR2 signaling is activated in this population of cells during inflammation. We used another reporter mouse line, CXCR4::EGFP, in which the transcription factors that normally regulate the expression of *cxcr4* gene additionally drive the expression of EGFP [17]. This mouse represents a transcriptional reporter, and unlike the protein level reporter CCR2-EGFP, CXCR4-expressing cells contain EGFP in the cytosol regardless of the status of CXCR4 activation (Fig 2 inset). CXCR4-positive cells include many cell types, including hematopoietic stem cells and monocytes. We challenged CCL2::CCL2-mRFP; CXCR4::GFP double transgenic mice with LPS and visualized whether CCL2 released from the stromal cell during inflammation acts on CXCR4-expressing cells (Fig 2A–2C). Indeed, clear CCL2-mRFP signal was visible inside some of CXCR4-positive cells only when the animal was injected with LPS (Fig 2B, yellow arrow; Fig 2B, red arrow), indicating that these cells co-express CCR2 and are likely to be CCR2/CXCR4 double positive monocytes. In contrast to CCL2::CCL2-mRFP; CCR2::CCR2-EGFP mice, in which most CCR2-positive cells contained intracellular CCL2-mRFP after LPS injection (Fig 1D and 1F), a smaller portion of CXCR4-positive cells showed internalized CCL2-mRFP, and these cells were in close proximity to CCL2-positive stromal cells (Fig 2B). To understand how the cells that co-express CCR2 and CXCR4 interact with CXCL12, the ligand for CXCR4, we crossed another transgenic mouse line, CXCL12::CXCL12-mRFP [7, 15] with CCR2::CCR2-EGFP (Fig 2D–2F). CCR2-positive cells (green arrow) were also juxtaposed with CXCL12-expressing cells (red arrow), which had elongated morphology similar to CCL2-positive cells (Figs 1B and 2E, red arrows). Upon LPS injection, CCR2 proteins become punctate, indicating CCR2 activation (Fig 2E, white arrow). These puncta never contained any CXCL12, indicating that CCR2-positive cells that are in close proximity to CXCL12-expressing cells were activated by endogenous CCL2 that are not labelled in this mouse line (Fig 2J). LPS injection did not induce activation of CXCR4 (i.e. internalization of CXCL12 by CXCR4-positive cells) (Fig 2G–2I). Consistent with this result, CXCL12-mRFP intensity was not changed by LPS injection indicating that its release is not affected by inflammation (S1C Fig). These results confirm that CCL2 released from the stromal cells act locally and activates CCR2 on nearby cells, which co-express CXCR4.

**Fig 3:**
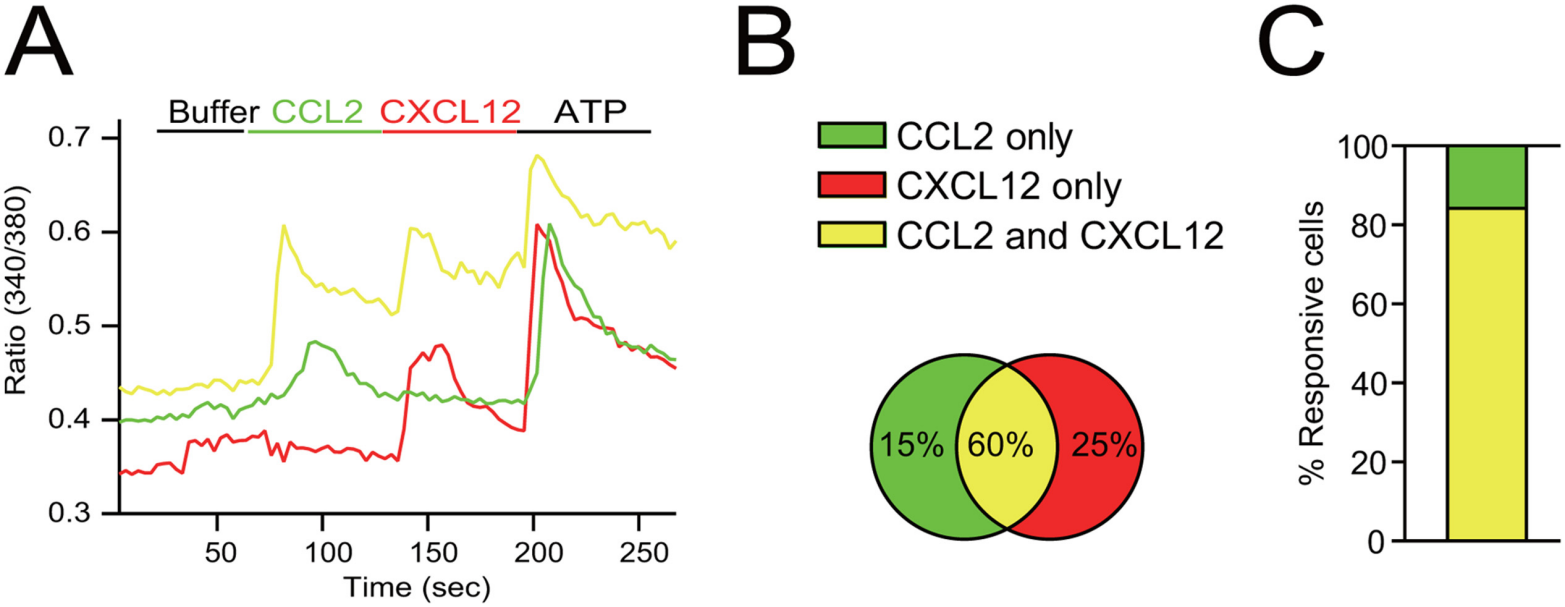
CCR2 and CXCR4 are co-expressed by bone marrow cells. (A) Acutely isolated bone marrow cells were plated on a cover slip and their responses to CCL2 (10 nM) and CXCL12 (10 nM) were measured by Fura 2-based calcium imaging. The response to ATP, which increases intracellular [Ca^2+^] by purinergic receptors, indicates that the recorded cell was alive. (B) Venn diagram showing percentage of CCL2- and/or CXCL12-responsive cells. (C) More than 80% of CCL2-responsive cells also responded to CXCL12.

Next, we examined whether these cells in the bone marrow co-express functional CCR2 and CXCR4 by performing Fura 2-based calcium imaging experiments on acutely isolated bone marrow cells. Activation of CCR2 and CXCR4 in most cells results in a transient rise in intracellular Ca2^+^ in a pertussis toxin-sensitive manner, which can be measured by calcium imaging [18, 19]. We treated acutely isolated bone marrow cells sequentially with vehicle solution, CCL2 and CXCL12 (Fig 3A), and categorized them according to their responses (Fig 3B). Most CCR2-positive cells (which responded to CCL2) also responded to CXCL12, indicating that the majority of CCR2-positive cells in the bone marrow indeed co-express functional CXCR4 (Fig 3C).

### 3. Activation of CCR2 desensitizes CXCR4 signaling which normally retains monocytes

**Fig 4:**
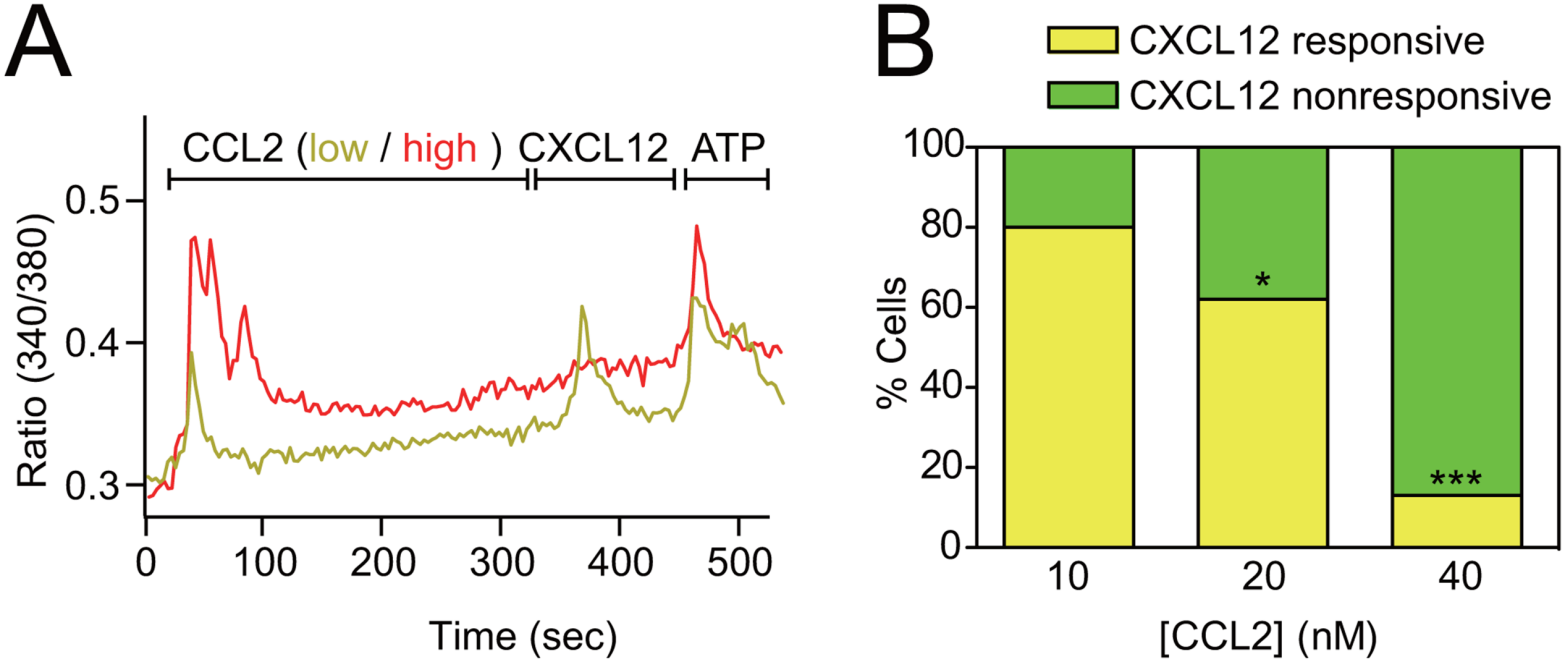
CCR2 desensitizes CXCR4. (A) Calcium imaging on acutely isolated bone marrow cells. A low (10 nM) or high (40 nM) of CCL2 was treated for 5 min before a CXCL12 application. ATP-responsiveness at the end of experiment shows cell viability. As shown in Fig 3, most CCL2-responsive cells also responded to CXCL12 (yellow). When a higher dose of CCL2 was pre-treated, however, most cells did not respond to CXCL12 (red). (B) Quantification of calcium imaging. n = 50, 95, and 30, respectively; *p<0.05 vs. 10 nM, ***p<0.001 vs. 10 nM or 20 nM; Fisher’s exact test.

**Fig 5:**
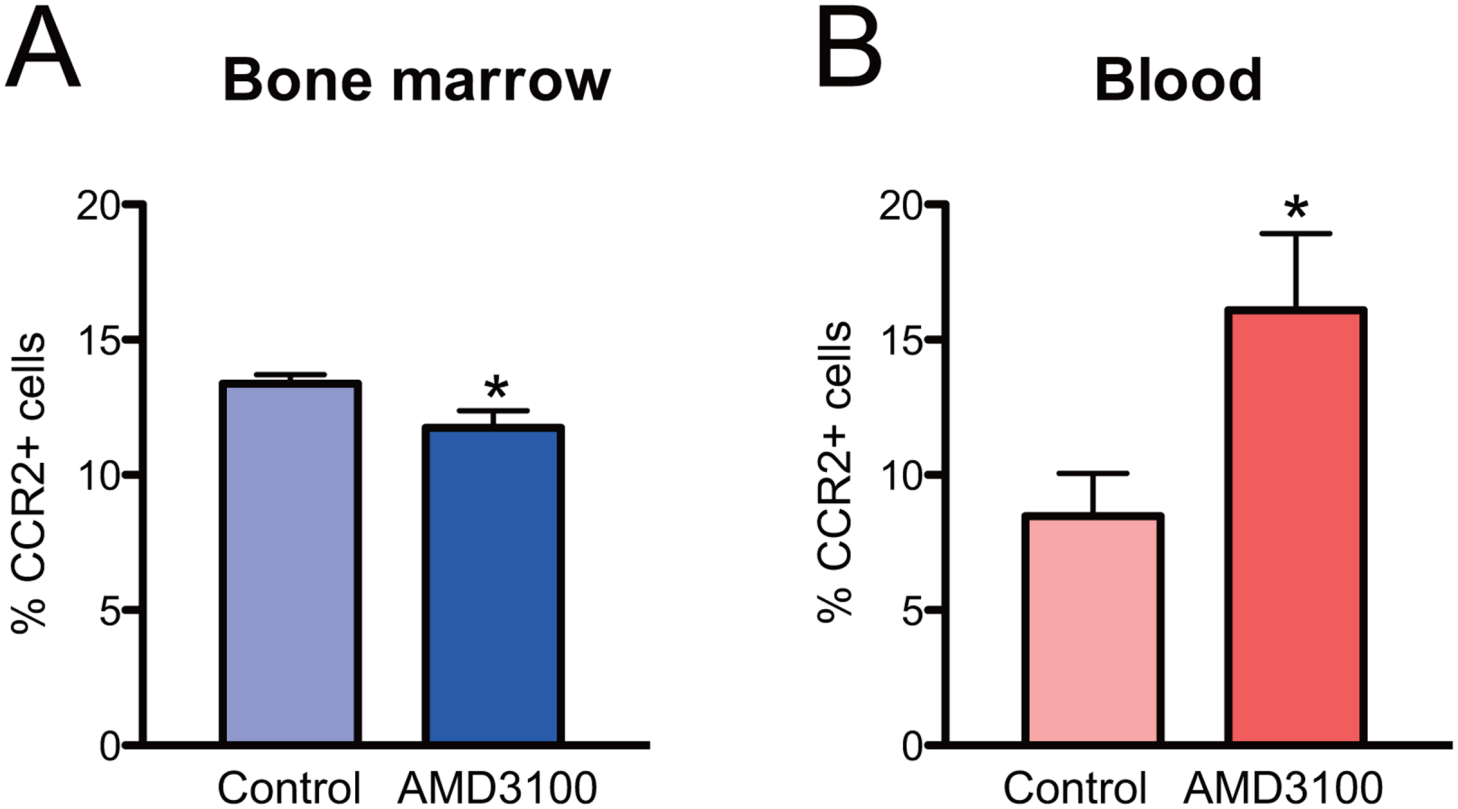
CCR2-positive cells can be mobilized by CXCR4 inhibition. CCR2::CCR2-EGFP mice were injected with AMD3100 or saline, and the percentage of EGFP-positive cells in the bone marrow or in the blood was measured by flow cytometry. AMD3100 injections decreased CCR2-EGFP cells in the bone marrow (A), and increased them in the bloodstream (B). n = 4 each; **p<0.01; Mann-Whitney test.

Because CXCL12/CXCR4 signaling is a well known signal that anchors various cells in the bone marrow [11], we hypothesized that the activation of CCR2 may desensitize CXCR4 expressed by the same cells, resulting in their mobilization. First, we again used Fura 2-based calcium imaging to investigate whether CCR2 activation desensitizes CXCR4 signaling. Acutely isolated bone marrow cells were sequentially treated with CCL2 and CXCL12 as previously, but this time higher doses of CCL2 (20 or 40 nM) were used (Fig 4A). Indeed, cells that were pre-treated with CCL2 showed a dose-dependent reduction in their responsiveness to CXCL12, indicating the desensitization of CXCR4 in these cells (Fig 4B). Reduction of CXCR4 activity was likely to result from CXCR4 desensitization rather than inactivation, because these cells were able to respond to higher doses of CXCL12 (S3 Fig). This result supports our hypothesis and predicts that antagonizing CXCR4-mediated signaling would promote the emigration of CCR2-positive monocytes even in the absence of any inflammatory stimuli. We tested this possibility by injecting CCR2-EGFP mice with AMD3100, a CXCR4 antagonist, and measuring the percentage of CCR2-positive cells in the bone marrow and in the peripheral blood by flow cytometry. In accordance with our prediction, an acute intraperitoneal injection of AMD3100 resulted in a significant decrease in the number of CCR2-positive cells in the bone marrow (Fig 5A). In contrast, the number of circulating CCR2-positive monocytes in the circulating blood increased (Fig 5B), suggesting that antagonizing CXCR4 activity indeed is sufficient to mobilize CCR2-positive monocytes from the bone marrow.

## Discussion

Recent studies by Pamer and colleagues showed that CCL2-CCR2 signaling participates in the “release” of classical monocytes from the bone marrow rather than in their “recruitment” toward the peripheral target [8]. In CCR2 knockout mice, and to a lesser extent CCL2 knockout mice, classical monocytes do not enter the bloodstream efficiently and instead accumulate in the bone marrow after bacterial infection. In addition *ccr2-/-* monocytes transferred to the bloodstream travel normally to the infected spleen [8]. Bacterial infection causes a rise in TLR ligands in the circulation, which is delivered to the bone marrow through sinusoidal endothelium. As a result, TLRs expressed by nearby cells, which include CXCL12-abundant reticular (CAR) cells, are activated. And it was believed that these cells then “up-regulate” CCL2, which causes CCR2-positive monocytes to exit the bone marrow through an unknown mechanism.

Here we showed that most CCR2-positive cells (~80%) in the bone marrow co-express CXCR4 (Fig 3C). Co-expression CCR2 and CXCR4 is consistent with the fact that CAR cells, which constitutively secrete CXCL12 under normal conditions, is one of the major sources of CCL2 during inflammation [6]. However, our results indicate that these and other bone marrow resident stromal cells express a significant level of CCL2, which is not released but stored in the intracellular vesicles (Fig 1B). The premade and stored CCL2 is then rapidly released upon the activation of the inflammatory response and activates CCR2 expressed by monocytes in close proximity (Fig 1C and 1D; S1A Fig). Although CXCL12 is also expressed in the bone marrow stromal cells under normal conditions, its cellular localization is not changed by inflammation (Fig 2D–2I; S1C Fig) suggesting that CXCL12 may be constitutively secreted in contrast to CCL2 whose release is regulated by inflammation. This result is consistent with our previous finding that the release of CCL2 is regulated by the calcium-dependent regulated pathway in peripheral sensory neurons, whereas CXCL12 is constitutively released [20].

**Fig 6:**
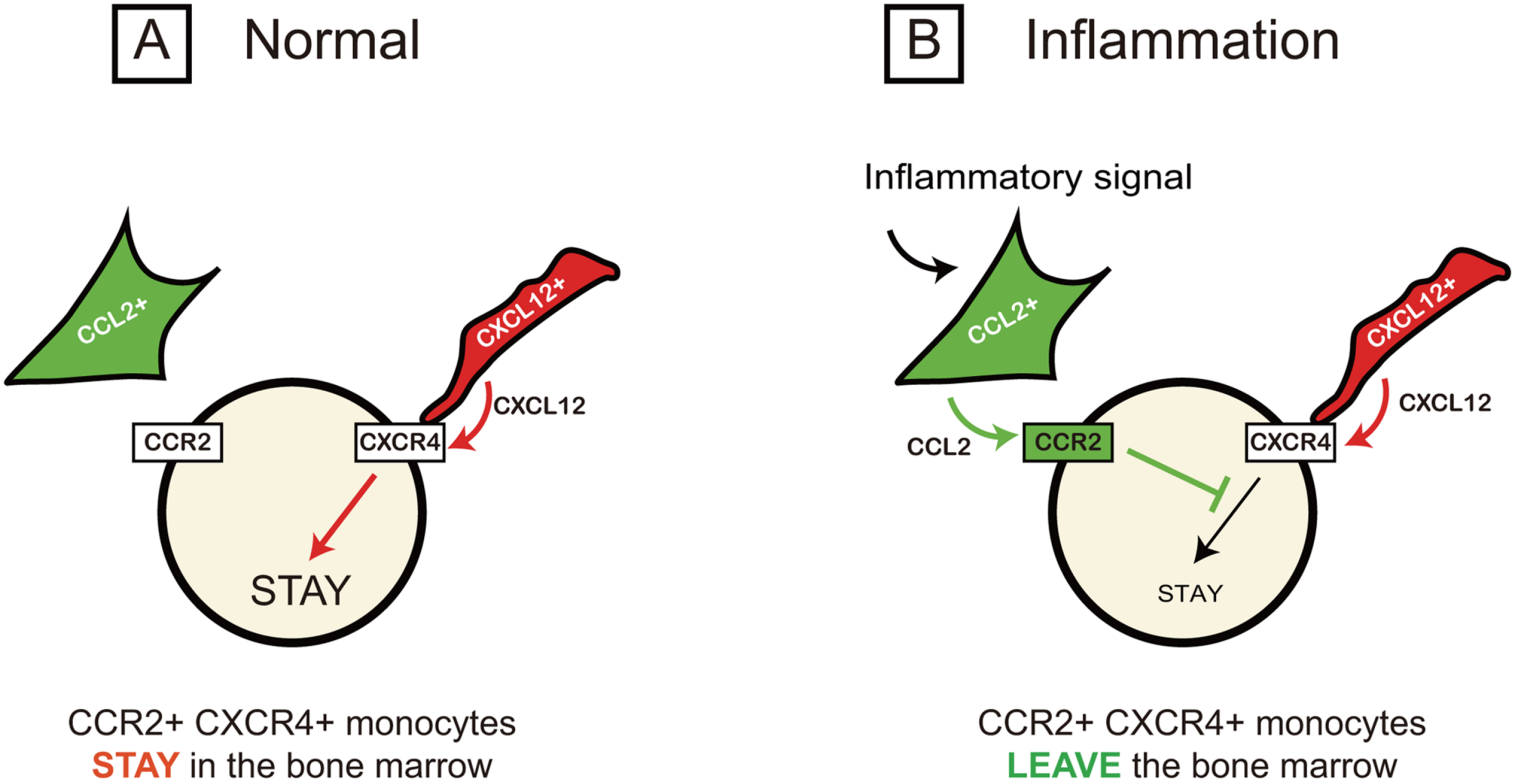
Proposed model: monocyte egress by CCR2-mediated cross-desensitization of CXCR4. Monocytes in the bone marrow co-express CXCR4 and CCR2. These cells are juxtaposed to bone marrow-resident cells, which express their respective ligands, CXCL12 and CCL2. (A) Under normal conditions, monocytes are retained by CXCR4 signaling, which is activated by constitutively secreted CXCL12. CCR2 on these monocytes is not active as its ligand CCL2 is stored but not released. (B) During inflammation, stored CCL2 is rapidly released and activates CCR2, which desensitizes CXCR4 and promotes cell migration.

It was unclear how CCL2, which is known to have a chemoattractant activity, promotes the migration of CCR2-positive monocytes away from the source of CCL2 gradient in the bone marrow. We showed that CCR2 and CXCR4 are co-expressed by the majority of bone marrow monocytes (Fig 3). We also showed that activation of CCR2 cross-desensitizes CXCR4 (Fig 4). Because CXCL12-CXCR4 signaling anchors and immobilizes the cell in the bone marrow, we hypothesize that CCL2-CCR2 signaling decreases this immobilization and increases overall mobility of the cells resulting in their migration out of the bone marrow (Fig 6). In accordance with our hypothesis, the classical monocytes identified by an independent way (CD11b+ Ly6Chigh) was also mobilized by AMD3100 (S4A Fig). Interestingly, AMD3100 did not mobilize the nonclassical monocyte subset (CD11b+ Ly6Clow) (S4C Fig) in contrast CCR2-positive classical monocytes (S4B Fig), suggesting that these cells might be retained by additional signals. Indeed, the nonclassical monocyte subset expresses a high level of CX3CR1, which was recently shown to retain cells in the bone marrow [21]. Therefore, CX3CR1 may provide an additional anchoring signal to the nonclassical but not classical monocytes.

Monocytes express not only CCR2 but also other chemokine receptors such as CCR5 [22], and CCR2 can be activated not only by CCL2 but also by CCL7 [1]. Other chemokines that act on CCR2 and other chemokine receptors may also regulate CXCR4 signaling on monocytes and their egress. CXCL12, which provides an anchoring signal to CXCR4-positive monocytes, not only binds to CXCR4 but also to CXCR7 [1]. CXCR7 can function as a scavenger for CXCL12 to regulate the availability of CXCL12 to CXCR4. Although leukocytes generally do not express CXCR7 [23], bone marrow-resident mesenchymal stem cells do [24]. Therefore, it is also possible that CXCR7 may regulate monocyte egress by regulating the concentration of CXCL12 in the bone marrow.

One potential mechanism for CCR2-mediated CXCR4 desensitization is direct interactions between receptors. Indeed, GPCR family members exhibit extensive homo- and hetero-oligomerization, and it has been shown that CCR2 and CXCR4 form heterodimers [25, 26]. Because CCL2 binding induces extensive endocytosis of cell surface CCR2 both *in vitro* [27] and *in vivo* ([28] and this paper), it is likely that a surge of CCL2 release from the bone marrow-resident stromal cells induces internalization of CCR2-CXCR4 complexes in juxtaposed monocytes and weakens the anchoring signal provided by constitutively released CXCL12. Another possibility is crosstalk between receptor-coupled intracellular signalling pathways. In this case, other GPCRs may also regulate CXCR4 activity and monocyte egress. In this regard, it is intriguing that CXCR4 hetero-oligomerize with α-adrenergic receptor in the vascular smooth muscle [29]. Because bone marrow progenitor cells express α-adrenergic receptors [30], our model may be extended to partially explain sympathetic control of the innate immunity [31].

Overall, our results explain how a rapid egress of bone marrow-resident monocytes is controlled by chemokine receptor crosstalk in the bone marrow niche where stromal cells and leukocytes form intricate networks, and suggest the antagonism of CXCR4 signaling as a promising therapeutic approach to treat monocytopenia. Indeed, WHIM (warts, hypogammaglobulinemia, infections, and myelokathexis) syndrome, an autosomal dominant disorder often accompanied by monocytopenia, is caused by a single amino acid mutation in CXCR4, which blocks its endocytosis and therefore increases CXCR4 activity [32]}. Therefore, inhibiting CXCR4 signaling could also be therapeutic for other diseases characterized by immobile monocytes.

## Materials and Methods

### Animal

All animal experiments were approved by the Institutional Animal Care and Use Committee at Yonsei University College of Medicine and Northwestern University. Animals were housed with food and water ad libitum and kept on 12-hours light cycle.

The following reporter transgenes were used: (1) CCR2::CCR2–EGFP (protein level reporter), (2) CCL2:CCL2–mRFP (protein level reporter), (3) CCL2::mRFP (transcriptional reporter), (4) CXCL12::CXCL12-mRFP [7], and (5) CXCR4::EGFP (transcriptional reporter) (GENSAT project, Rockefeller University, New York, NY). Mice were injected intraperitoneally with 10 mg/kg lipopolysaccharide (LPS) diluted in saline and were killed after 6 hours.

### Histology and imaging

For tissue sections, lumbar vertebrate columns were immediately removed from mice transcardially perfused with 4% paraformaldehyde and post-fixed for 4 h. The tissues were sectioned at 14 μm and stored at -20°C until use. Images were taken in a laser-scanning confocal microscope (Olympus, Melville, NY, USA) equipped with a 60x oil immersion objective lens (numerical aperture, 1.40). For quantitative analysis, the tissue samples to be compared were prepared together in identical conditions. Then the images were taken with identical acquisition settings. All images used for quantitative analysis covered 200μm x 200μm x 10μm per field, which was taken by 1 μm-thick optical section in 1 μm interval. For cell counting, we used ImageJ plugin Cell Counter. We went through individual optical sections for accurate measurement of cell numbers per field. For RFP intensity measurement, we first made a Z-projection using ImageJ and then made ROIs by manually drawing cell boundaries. We only analyzed non-overlapping cells. For 3D reconstruction, we used ImageJ plugin 3D Viewer.

### Bone marrow cell isolation and flow cytometry

CCR2::CCR2-EGFP mice were injected intraperitoneally with either AMD3100 (10mg/kg) or saline at 8 am. Two hours later mice were anesthetized, both blood and bone marrow were harvested using standard methods. Single cell preparations were then counted and re-suspended in FACS buffer (PBS with 0.1% BSA) to 1x10^6^ cells/ml. The following antibodies were used for multiple fluorescence labeling, all from Becton Dickinson: CD11b-APC and Ly6C-PE. CCR2-EGFP occupied the GFP channel, and propidium iodide was added to each sample. Single channels were used for compensation adjustment then multi-color fluorescence was acquired on a Becton Dickinson flow cytometer. Initially CCR2-EGFP+ cells were identified from the population of viable cells. To examine Ly6C+/CCR2+ monocytes, the gating strategy employed involved selecting CD11b+ cells followed by examining the Ly6C/CCR2 co-labeled population.

### Calcium imaging

The AM form of fura-2 (Invitrogen) was used as the fluorescent Ca^2+^ indicator. All measurements were made at room temperature (21°C) as described previously (Bhangoo et al. 2007). Acutely isolated bone marrow cells loaded with fura-2AM (3 μM) were mounted onto the chamber (500 μL total volume), which was then placed onto the inverted microscope, and perfused continuously by balanced salt solution (in mM: 145 NaCl, 5 KCl, 2 CaCl_2_, 1 MgCl_2_, 10 N-2-hydroxyethylpiperazine-N′-2-ethanesulfonic acid, and 10 glucose). Ratiometric images were collected every 3 s and Ca^2+^ concentration was calculated by a standard curve generated using calcium calibration buffer kit (C-3721, Invitrogen) using MetaFluor software (Molecular Devices). For CCL2 and CXCL12 (R & D Systems, Minneapolis, MN, USA), we applied 1 mL of solution directly to the bath chamber after stopping the flow.

### Statistics

Data were presented as mean ± SEM. Mann-Whitney test or Fisher’s exact test were performed as described in the figure legend. Statistical significance was set at p < 0.05.

## Supporting Information

S1 Fig LPS induces the release of stored CCL2 in the bone marrow. (A) Normalized net RFP intensity per cell was measured in CCL2::CCL2-mRFP mouse (CCL2 protein level reporter) injected with LPS or vehicle. LPS injection significantly decreased RFP intensity per cell (which correlates with CCL2 contents), indicating CCL2 release. (B) RFP intensity did not change in CCL2::mRFP (transcriptional reporter). (C) CXCL12-mRFP release was not affected by LPS. The numbers indicate the numbers of analyzed cells taken from three independently performed experiments.
(EPS)

S2 Fig The numbers of CCL2- and CCR2-positive cells in the bone marrow. (A-B) CCL2- and CCR2-positive cells were counted in optical sections with the dimension of 200μm x 200μm x 10μm. LPS injection increased in CCL2-positive cells and decreased CCR2-positive cells in the bone marrow. (A) CCL2::mRFP. (B) CCR2::CCR2-EGFP. n = 4; *p<0.05; Mann-Whitney test.
(EPS)

S3 Fig CCR2 desensitizes CXCR4. Quantification of calcium imaging on acutely isolated bone marrow cells. Cells were treated with CCL2 for 5 min and then with CXCL12 with indicated concentrations. n = 22, 10, 63, 30, respectively; ***p<0.001 vs. 20 nM CCL2 / 20 nM CXCL12 group; Fisher’s exact test.
(EPS)

S4 Fig AMD3100 mobilizes the classical monocytes. (A-C) Quantification of the classical (CD11b+Ly6Chigh) and nonclassical (CD11b+Ly6Clow) monocyte subsets by flow cytometry. CCR2::CCR2-EGFP mice were injected with AMD3100 or saline, and the percentage of EGFP-positive cells in the bone marrow or in the blood was measured by flow cytometry. AMD3100 decreased the classical monocytes in the bone marrow and increased them in the bloodstream (A, B). AMD3100 did not change distribution of the nonclassical monocytes (C). n = 3; **p<0.01; Mann-Whitney test.
(EPS)
